# Geometric Correction for the Geostationary Ocean Color Imager from a Combination of Shoreline Matching and Frequency Matching

**DOI:** 10.3390/s18113599

**Published:** 2018-10-23

**Authors:** Han-Gyeol Kim, Jong-Hwan Son, Taejung Kim

**Affiliations:** 1 3DLabs Co. Ltd., 100 Inharo, Michuhol-Gu, Incheon 22212, Korea; khanai@3dlabs.co.kr; 2 Department of Geoinformatic Engineering, Inha University, 100 Inharo, Michuhol-Gu, Incheon 22212, Korea; json8520@gmail.com

**Keywords:** GOCI, geometric correction, sensor modeling, shoreline matching, frequency matching

## Abstract

Geometric correction is fundamental in producing high quality satellite data products. However, the geometric correction for ocean color sensors, e.g., Geostationary Ocean Color Imager (GOCI), is challenging because the traditional method based on ground control points (GCPs) cannot be applied when the shoreline is absent. In this study, we develop a hybrid geometric correction method, which applies shoreline matching and frequency matching on slots with shorelines and without shorelines, respectively. Frequency matching has been proposed to estimate the relative orientation between GOCI slots without a shoreline. In this paper, we extend our earlier research for absolute orientation and geometric correction by combining frequency matching results with shoreline matching ones. The proposed method consists of four parts: Initial sensor modeling of slots without shorelines, precise sensor modeling through shoreline matching, relative orientation modeling by frequency matching, and generation of geometric correction results using a combination of the two matching procedures. Initial sensor modeling uses the sensor model equation for GOCI and metadata in order to remove geometric distortion due to the Earth’s rotation and curvature in the slots without shorelines. Precise sensor modeling is performed with shoreline matching and random sample consensus (RANSAC) in the slots with shorelines. Frequency matching computes position shifts for slots without shorelines with respect to the precisely corrected slots with shorelines. GOCI Level 1B scenes are generated by combining the results from shoreline matching and frequency matching. We analyzed the accuracy of shoreline matching alone against that of the combination of shoreline matching and frequency matching. Both methods yielded a similar accuracy of 1.2 km, which supports the idea that frequency matching can replace traditional shoreline matching for slots without visible shorelines.

## 1. Introduction

Images taken from satellites contain many geometric distortions. Geometric distortions are caused by various factors, such as the complex arrangement of image sensors, position errors between image bands, the Earth’s curvature and rotation, satellite’s position and attitude sensor error, and surface altitude variations. Geometric correction to remove such distortions must be performed for the production of spatial information. To correct geometric distortions, a precise sensor model should be established, and an appropriate sensor modeling technique should be applied, according to the characteristics of the satellite images.

Currently, various geometric correction schemes utilize ground control points (GCPs) for precise sensor model establishment. For geostationary satellites such as GOES-9 and MTSAT-1R, geometric correction includes an image navigation process. The image navigation process establishes precise sensor orientation by matching the image with shorelines and by using the matched results as GCPs [1].

**Figure 1:**
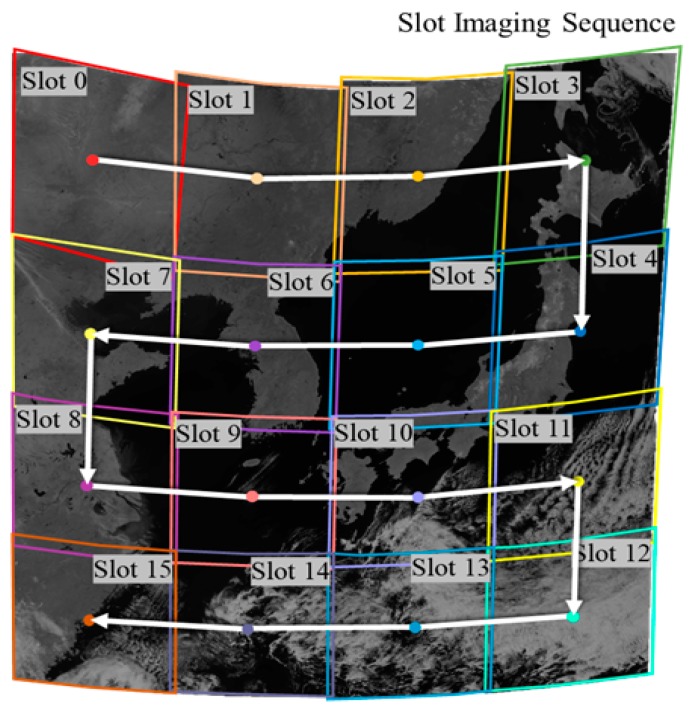
GOCI slot imaging sequence.

The Geostationary Ocean Color Imager (GOCI) is an image sensor mounted on a geostationary satellite, GK-1, and it takes images over an area of 2500 km by 2500 km through 16 consecutive slots. See Figure 1 for the GOCI coverage and imaging sequence. Each slot contains a different amount of geometric distortion due to Earth’s curvature and the platform’s movement between slots. It is necessary to remove geometric distortion within each slot, and then mosaic the 16 geometrically corrected slots into one GOCI Level 1B scene for further analysis and utilization. In general, GOCI geometric correction is performed by matching image slots with shorelines [2]. However, some GOCI slots cover ocean areas without any shorelines. Some slots may show heavy cloud, and the shorelines may not be visible. It is not possible to utilize shoreline-based geometric correction for all GOCI slots. In this paper, we try to perform geometric correction of GOCI images by applying shoreline-based geometric correction to the slots with visible shorelines, and by estimating the relative orientation between adjacent slots for the slots without shorelines.

Estimation of the relative orientation between two images requires tie-points (image points of common features observable in both images). Tie-points are commonly extracted from feature points, which can be detected by algorithms such as Scale Invariant Feature Transform (SIFT), Speeded Up Robust Features (SURF), and Oriented FAST and Rotated BRIEF (ORB) are commonly used to extract tie-points based on feature points [3,4,5]. However, the feature-based tie-point extraction method cannot be applied to the GOCI slots, which may contain little or no texture information. This issue can be addressed by frequency domain matching for extracting tie-points and estimating the relative orientation of GOCI slots [6]. Frequency domain matching transforms images into the frequency domain through Fourier transform, extracts a correlation peak of the two images by multiplying the frequency power spectrum of the two images, and estimates image translation between the two images by transforming the peak into the spatial domain [7,8,9]. This method has been used for image alignment with a translational relationship [10] and was extended to handle rotational and similar transformations [11].

In this paper, we extend our earlier research [6] to combine frequency matching results with shoreline matching results for absolute orientation and geometric correction in GOCI images. We describe the proposed method of GOCI geometric correction in Section 2. Section 3 introduces our experiment dataset, and Section 4 reports results and offers analysis. Finally, Section 5 describes the conclusion of this paper and future plans.

## 2. Proposed Method

**Figure 2:**
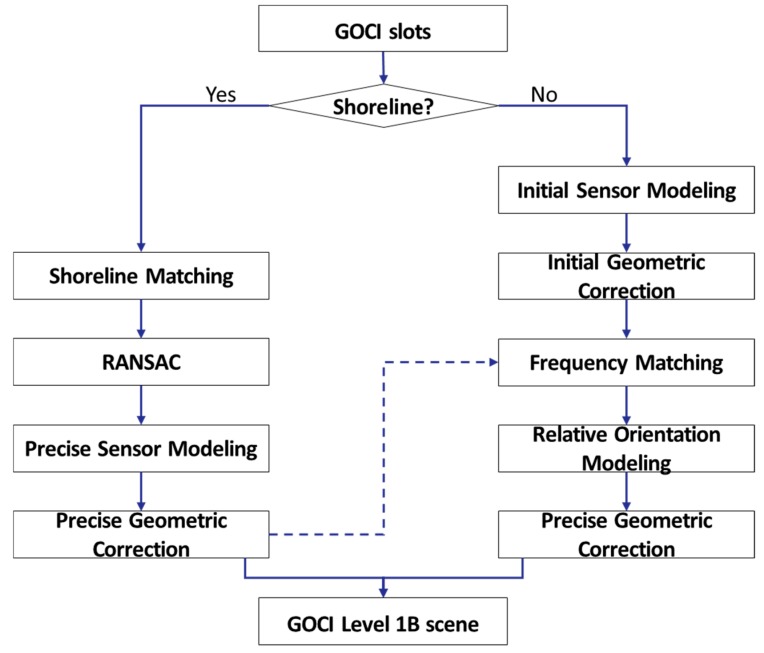
Process of the proposed method.

GOCI slots can be divided into cases where shorelines are visible and where they are not. For the slots with shorelines, image navigation based on shoreline matching can be used for geometric correction. For slots without a shoreline, geometric correction can be performed by estimating transformation relationships with adjacent slots through frequency matching. Figure 2 shows the process in our proposed method. 

### 2.1. Initial Sensor Modeling and Geometric Correction

**Figure 3:**
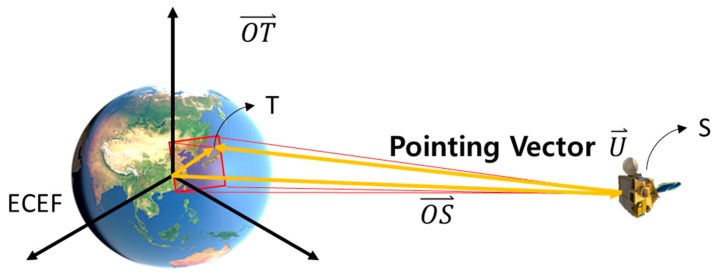
Sensor modeling concept.

Sensor modeling is a process that establishes the geometric relationship between the image coordinate system and the ground coordinate system when the image was shot. Figure 3 shows a diagram of sensor modeling.

Each image pixel generates a pointing vector, $\vec{U}$, from the sensor (S) to a ground point (T). The relation between the sensor position ($\vec{OS}$), ground point vector ($\vec{OT}$), and pointing vector $\vec{U}$ can be expressed as

(1) $$\vec{OT}=\vec{OS}+\lambda \vec{U}$$

where λ is a scale factor.

Geometric correction is a process that relocates image pixels according to the ground coordinate frame. In this paper, sensor modeling and geometric correction processes are divided into initial correction and precise corrections. Initial sensor modeling establishes the geometric relation using only the ephemeris information provided by the satellite’s on-board sensors when shooting the images, and initial geometric correction relocates the original image accordingly. The ephemeris information may include errors, and these errors affect the accuracy of sensor modeling and geometric correction. Therefore, it is necessary to remove these errors, and this process is precise sensor modeling. Precise geometric correction refers to image relocation using a precise sensor model. 

In the proposed method, all slots without shorelines undergo initial sensor modeling and initial geometric correction. Through these, distortions due to Earth’s curvature can be removed. 

### 2.2. Shoreline Matching and Precise Sensor Modeling

For slots with shorelines, shoreline matching and precise sensor modeling are performed. Matching results between shorelines and the GOCI slot are used as GCPs. Since the performance of the GCPs greatly affects precision sensor modeling, the accuracy in shoreline matching is very important. 

**Figure 4:**
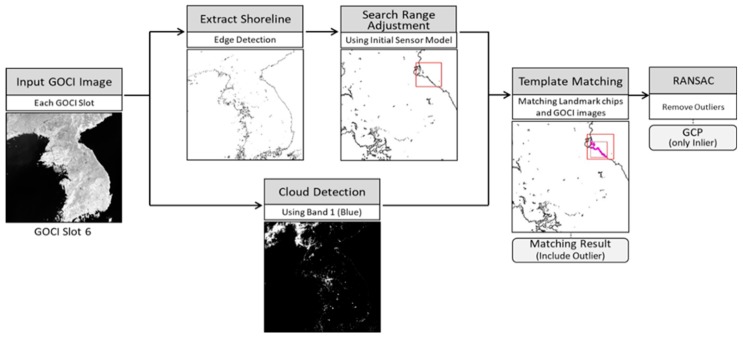
Shoreline matching process.

For shoreline matching, we use GOCI near infrared (NIR) band images. This is because reflectance differences between water and land are largest in the NIR band, leading to best edge detection. The overall process of shoreline matching is as follows (Figure 4). Firstly, we extract the shoreline from GOCI slots through edge detection. We also construct shoreline chips by projecting a shoreline database into the GOCI image geometry using the GOCI initial sensor model. Secondly, using the initial sensor model, we adjust the search region to reduce outliers and improving matching speed. The center of the search region is calculated by the initial sensor model, and by considering the error range of the initial sensor model, the search region is set as 75 × 75 pixels. On the other hand, in order to prevent outliers in the areas occluded by thick clouds, we detect thick cloud using reflectance of the GOCI blue band [12]. If the detected cloud within the search range is over 20%, we skip the matching. If not, we conduct matching between shoreline chips and the edge-filtered GOCI NIR band. Through the matched results, we obtain image coordinates corresponding to ground coordinates of shoreline chips. These matching results may include outliers, and we remove them using random sample consensus (RANSAC) [13]. Finally, we use these matched results without outliers as GCPs.

RANSAC estimates a model with the minimum required number of measurements data selected randomly and checks whether other measurement data support the model. It repeats this process for a certain number of time and chooses the model with the largest supports [13]. RANSAC is a powerful robust estimator that is used in many applications. It works as far as there exists a model to formulate measurement data and one can postulate a boundary between inliers and outliers [14].

Precise sensor modeling is performed by updating three Euler angles of the pointing vector. Using GCPs, we calculate the angles, which correct position and rotation errors from the initial sensor model. According to ground coordinates calculated by the precision sensor model, we resample all image pixels to make a GOCI Level 1B scene.

### 2.3. Frequency Domain Matching and Relative Orientation Modeling

Since it is not possible to extract GCPs for slots without shorelines, we establish relative orientations between slots without shorelines and adjacent slots with shorelines through frequency matching.

**Figure 5:**
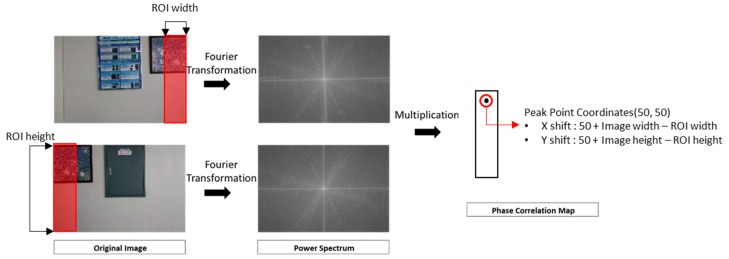
Process of Fourier transformation and phase correlation.

Two images are converted to frequency power spectrums through Fourier transformation. The two power spectrums are then multiplied to produce a phase correlation map. In a phase correlation map, values indicate the correlation between two images, and the positions represent the amount of shift between two images. For matching two images, the shift between them is determined using the position of the peak point in the phase correlation map. Figure 5 shows an example of the frequency matching process. In the example, the position of the peak value in the correlation map is (50, 50), and the relative shift can be calculated using these coordinates, the size of the image, and the region of interest (ROI).

**Figure 6:**
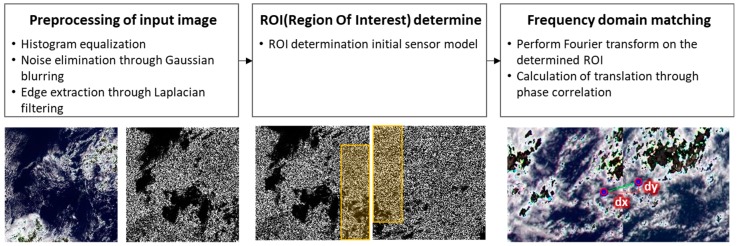
Process of frequency domain matching for GOCI slots.

For frequency matching of GOCI slots, we can increase the success rate through image preprocessing and precise-matching region selection [6]. This method is shown in Figure 6.

For the image preprocessing step, histogram equalization, Gaussian blurring, and Laplacian filtering are performed on two images. Since signals recorded in GOCI slots are mostly from the ocean, local variation of signals or image texture may not be large. On the other hand, a larger signal variation is favorable in frequency matching. To handle this situation, we applied histogram equalization and a Laplacian filter to amplify the rate of change in the brightness value of the image and show that this can increase the success rate with frequency matching. Gaussian blurring is performed before Laplacian filtering because Laplacian filtering is sensitive to noise.

Matching the ROI between neighboring slots is precisely determined by calculating the overlapping area through initial sensor models. Fourier transformation and frequency matching are applied only to the ROIs. We showed that this could shorten the processing time and improve the success rate of frequency matching.

**Figure 7:**
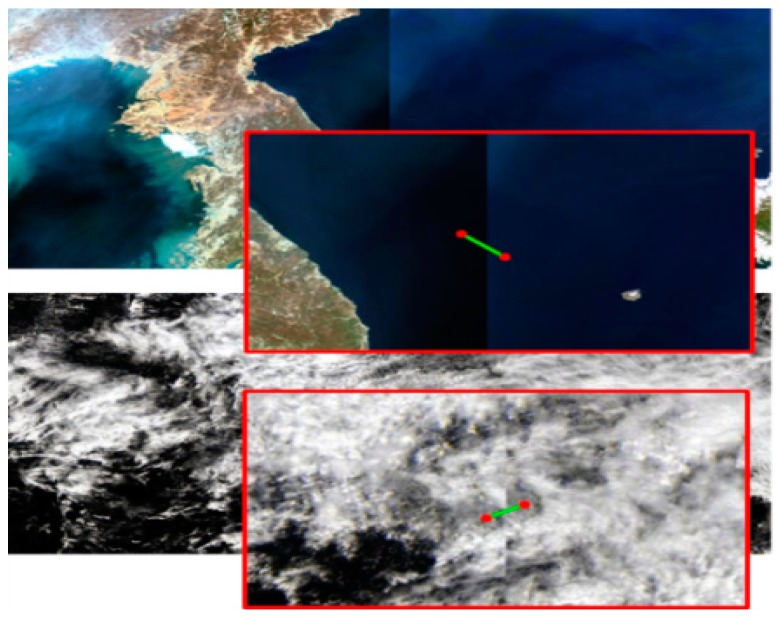
Frequency domain matching result of oceanographic images.

Through this process, the amount of parallel shift between two GOCI slots can be calculated. Figure 7 shows an example of frequency matching between two neighboring GOCI slots.

### 2.4. Precise Geometric Correction and GOCI L1B Generation

After frequency matching, we perform precise geometric correction for the slots without shorelines. The following two assumptions are applied.

- As a result of the initial sensor modeling, the geometric correction for earth-curvature and rotation is completed.
- The transformation relationship between adjacent slots is regarded as a rigid transformation.

The first assumption is valid when the initial model is close to the true model and the remaining errors can be removed by very small amount of Euler angles. It is known that very small amount of angular transformation over a small plane can be approximated by translation. The second assumption is valid when time difference between adjacent slots is not significant.

From the above two assumptions, the transformation relationship from frequency matching is applied to the initial geometric correction result for precise geometric correction.

**Figure 8:**
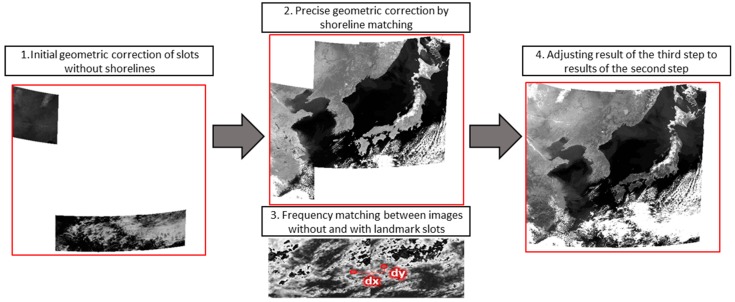
Process of mosaicking under our method.

The overall image mosaicking process is shown in Figure 8. Initial geometric correction is performed on slots without shorelines. Next, precise geometric correction is performed on slots with shorelines through shoreline matching. Frequency matching is performed between a slot without shorelines and an adjacent slot with shorelines. Lastly, the shift obtained from frequency matching is applied to the initial geometric correction result.

## 3. Experiment Dataset

**Figure 9:**
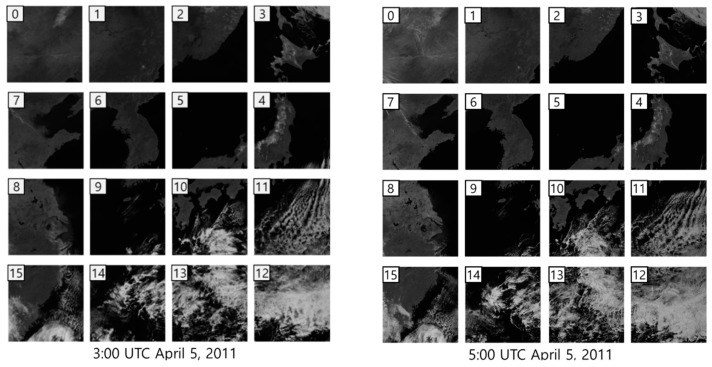
Experimental data (GOCI Level 1A).

We used eight GOCI Level 1A datasets, which were photographed from 00:00 to 07:00 UTC 5 April 2011, for the experiments. Figure 9 shows examples of 16 GOCI slots in the NIR band.

**Figure 10:**
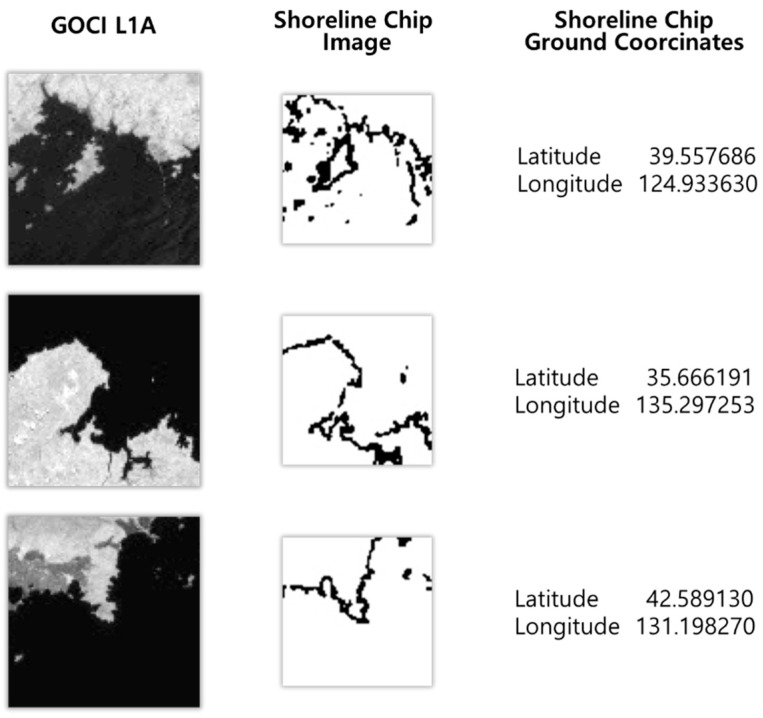
Examples of shoreline chips.

To perform shoreline matching, we constructed a shoreline chip database. We used the Global Self-consistent, Hierarchical, High-resolution Geography (GSHHG) database as raw data in the shoreline chip database [15]. The GSHHG database was constructed from three public domain shoreline databases: World Data Bank II (WDB II), World Vector Shoreline (WVS), and Atlas of the Cryosphere (AC) [16]. We projected the GSHHG database using the GOCI ephemeris to acquire shoreline images that match the GOCI images and constructed the shoreline chip database by extracting small-image chips centered on dominant shorelines. We had analyzed the matching success rate according to specifications of the shoreline chip database. Based on the result, we constructed a shoreline chip database of images with an 81 × 81 pixel size, 1.5 km thickness, and 0.55 km spatial resolution [17]. As shown in Table 1, we constructed a total of 451 shoreline chips. In slots 0 and 1, a relatively small number of shoreline chips were constructed because there was less shoreline within those slots. Figure 10 shows examples of shoreline chips constructed and corresponding to GOCI slots.

**Figure 11:**
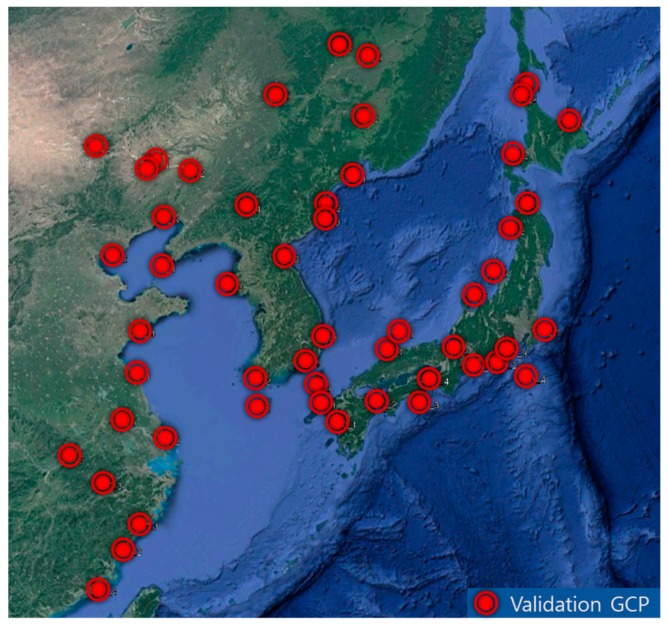
Experimental data (GOCI Level 1A).

**Figure 12:**
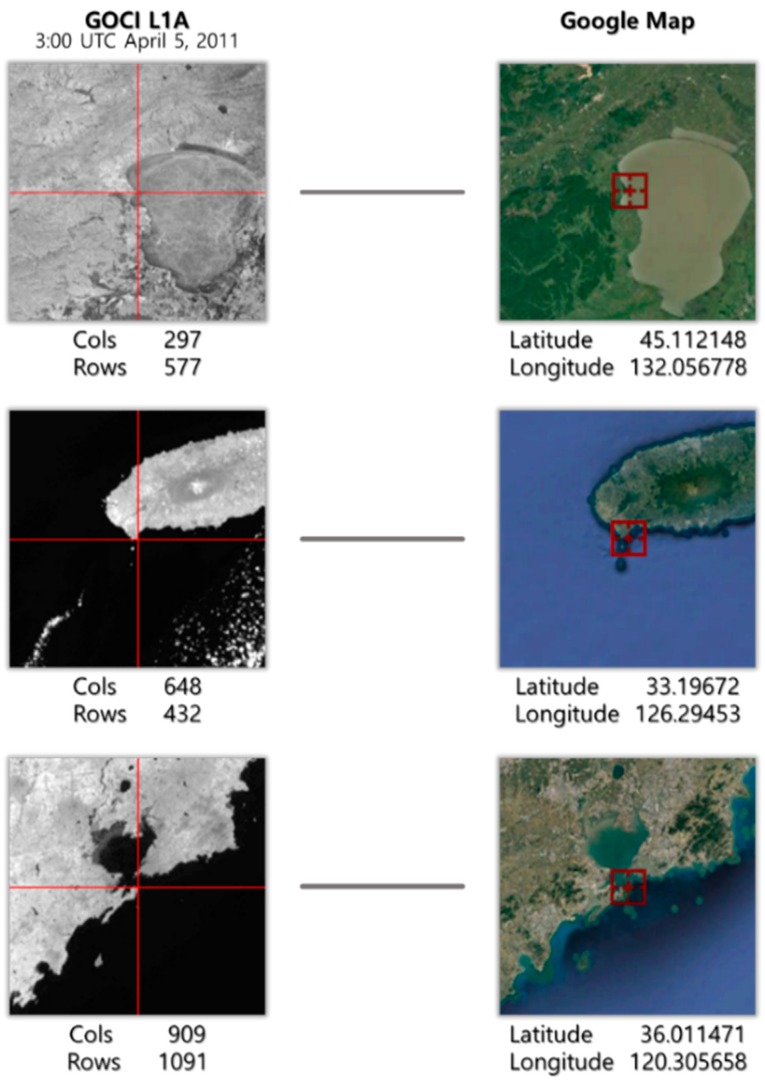
Examples of validation GCPs and the corresponding parts of GOCI slots.

In order to validate the quality of the geometric correction, we generated 52 validation GCPs by measuring precision ground coordinates using Google Maps. The GCPs were extracted so as to be distributed evenly on each slot. Validation GCPs were extracted by averaging measurements four times per slot, except for slots 12, 13, and 14. Figure 11 shows the distribution of the GCPs, and Figure 12 shows examples of the GCPs and the corresponding parts of the GOCI slots. The accuracy of Google Earth images is expected to have a horizontal error of about 1.8 m to 3.63 m and a vertical error of 1.73 m, on average [18]. Since the spatial resolution of GOCI is 500 m, it is possible to verify the geometric correction results through validation GCPs acquired through Google Earth.

## 4. Experiment Results and Analysis

In this paper, three experiments were conducted to verify the proposed method. In the first experiment, we checked the accuracy of general geometric corrections using shoreline matching. This experiment was performed to investigate the degree of geometric correction performance when shoreline matching is applied. In this experiment, slots 12, 13, and 14 were not used because they have no shorelines. The second experiment was conducted to check the performance of frequency matching. In this case, frequency matching was applied to the same slots as the first experiment. This was to compare the performance of frequency matching with that of shoreline matching. In the last experiment, we checked the performance of the proposed method for GOCI geometric correction by visual analysis.

### 4.1. Geometric Correction by Using Shoreline Matching

For slots with shorelines, we performed geometric correction by using shoreline matching. As mentioned earlier, shoreline chips of 81 × 81 pixels and a search range of 75 × 75 pixels were used. Table 2 shows the results from shoreline matching by showing all matched points (“Match” in the table) and the number of outliers within the matched points (“Outlier”). There were 15 to 37 outliers for each dataset.

**Figure 13:**
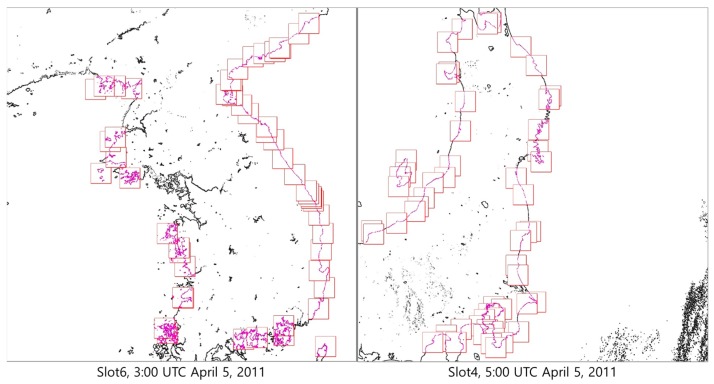
Shoreline-matching–result images (after RANSAC).

We applied RANSAC to the matching results. When we applied RANSAC based on the GOCI sensor model, all outliers were successfully removed. We can check the results in Table 3 by checking the number of inliers identified by RANSAC (“Inlier”) and the number of outliers within the inliers (“Outlier”). We used the inliers as GCPs for precise sensor modeling. Figure 13 shows an example of shoreline matching after RANSAC.

As shown in Table 4, the average root mean square error (RMSE) was 1.1 km in eight datasets. In slots 0 and 1, the geometric correction quality was worse than other slots, because the GCP number from shoreline matching was not sufficient.

### 4.2. Geometric Correction by Using Frequency Matching

Frequency matching was performed for all slots except 12, 13, and 14 (without shorelines), and the accuracy was measured. The geometric correction process used frequency matching with adjacent slots according to the GOCI imaging sequence. Frequency matching was performed once for slots 0, 11, and 15 and twice for the other slots. Table 5 shows the frequency matching results as before by showing the number of matched points (“Match”) and the number of outliers within the matched results (“Outlier”) with underlines. Outliers occurred at the maximum (3 times) for each dataset.

Table 6 shows the accuracy of geometric correction with shifts obtained as a result of frequency matching. It shows approximately 1.5 km errors for all slots, which is close to the error with shoreline matching. However, due to the outliers in frequency matching, the accuracy of the slots with outliers was large as underlined in Table 6. When the outliers were removed, the accuracy of these slots was improved greatly and the average RMSE for all slots was improved to 1.2 km, as shown in Table 7.

### 4.3. Geometric Correction with the Proposed Method

**Figure 14:**
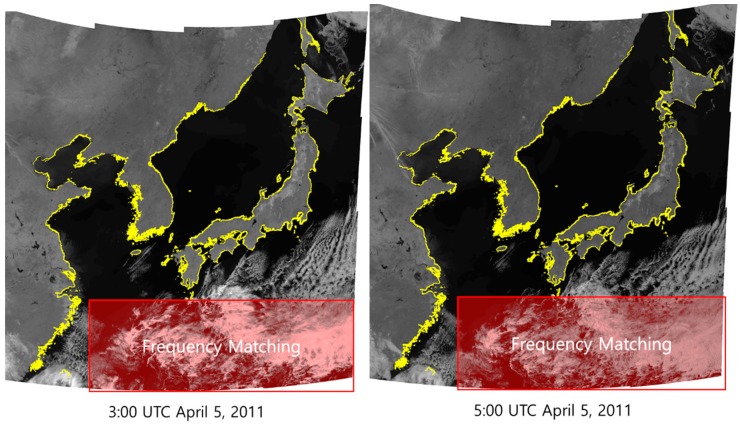
The result of the experiments.

For the final experiment, we applied the proposed method to all 16 GOCI slots. We classified slots 12, 13, and 14 as slots without shorelines. For slots with shorelines, we applied shoreline matching. For slots 12, 13, and 14, we applied frequency matching between slots 15-14, 14-13 and 13-12, sequentially. After all processing steps, we generated mosaic images. Figure 14 shows examples of mosaicked images with shorelines in yellow. We can see that the shorelines fit the images well.

**Figure 15:**
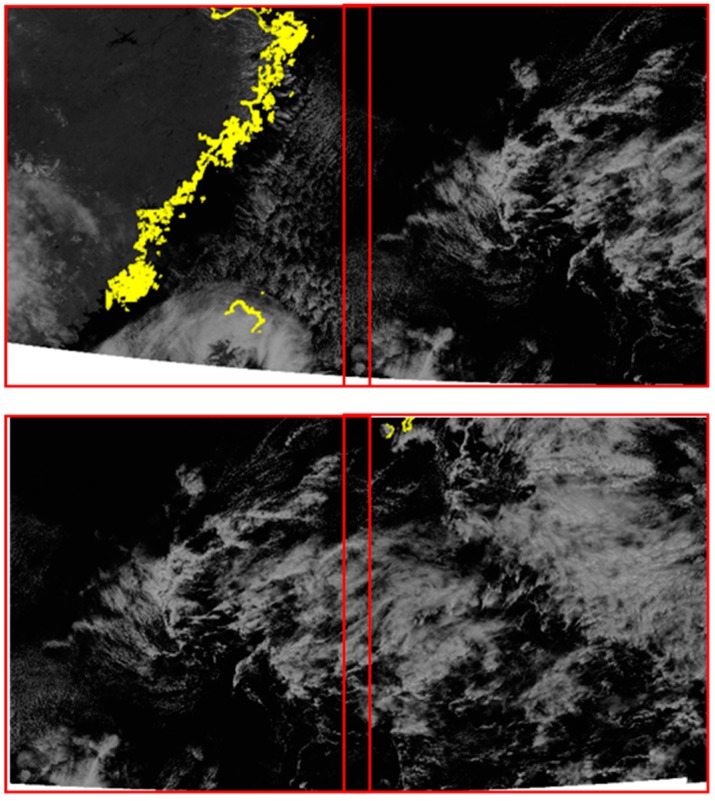

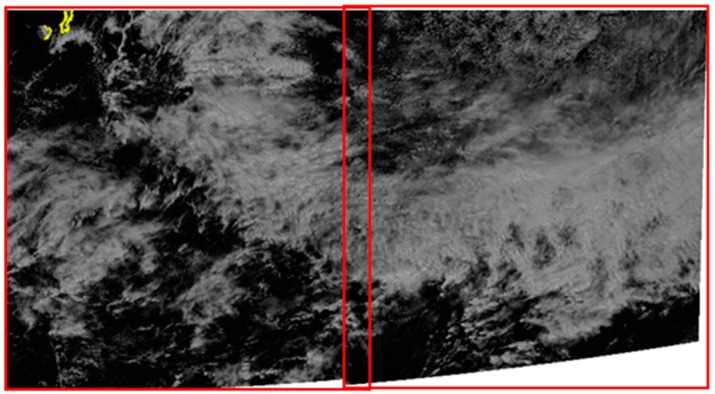
Boundary check between slots 15-14 (**top**), 14-13 (**middle**), and 13-12 (**bottom**).

We checked whether frequency matching was performed properly at the boundary of the slots where frequency domain matching was performed. Figure 15 shows mosaicked boundaries between slots 15-14, 14-13 and 13-12. We can see there are no visible seamlines between adjacent slots.

**Figure 16:**
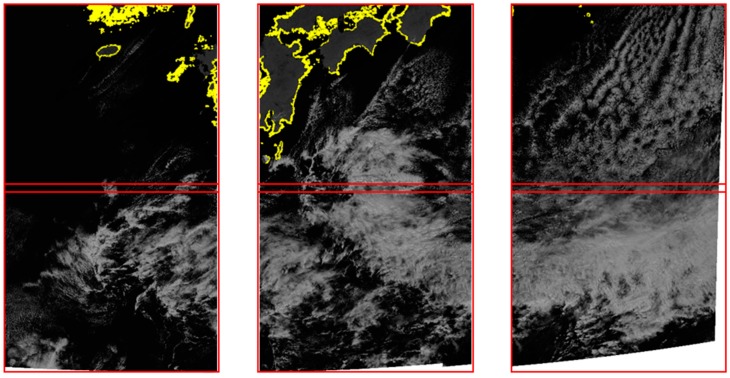
Boundary check between slots 14-9 (**left**), 13-10 (**middle**), and 12-11 (**right**).

We also checked further whether frequency matching could produce seamless boundaries between the slots processed by frequency matching and the slots processed by shoreline matching independently. Figure 16 shows mosaicked boundaries between slots 14-9, 13-10, and 12-11. Note that frequency matching was applied to slots 15-14, 14-13, and 12-13 and independent shoreline matching was applied to slots 9, 10, and 11. We can see there are no visible seamlines between adjacent slots. As a result, we confirmed the applicability of frequency domain matching to GOCI geometry correction.

### 4.4. Summary of All Experiments

In this section, we compare the accuracy of all three experiments in Table 4, Table 6 and Table 7. With frequency matching, accuracy was lower than shoreline matching. However, if outliers are removed, the performance is close to that of shoreline matching. In addition, it shows better performance than shoreline matching in slot 0, which had very few shoreline matching results. 

As mentioned before, the success of frequency matching is credited from accurate initial models so that large distortions and errors have been removed by initial geometric correction. Accurate initial models also played an important role in ROI determination for frequency matching. Nevertheless, our results showed that frequency matching can be a good candidate for geometric correction where shorelines are not visible.

## 5. Conclusions

In this paper, we proposed a combination of shoreline matching and frequency matching to perform geometric correction for GOCI data. This hybrid method can be applied to slots with or without shorelines. Our experimental results show a RMSE of 1.1 and 1.2 km for the shoreline matching and frequency matching (without outliers), respectively. Our results also suggest that frequency matching can accurately estimate translational relationships of GOCI slots.

Limitations of our experiments include that we considered one frequency matching between two adjacent slots and hence extracted one tiepoint. By defining multiple ROIs from the overlapping regions, we may generate multiple ties and apply more sophisticated transformation for geometric correction. This will be our future research. Another limitation could be the slot-based processing we used for geometric correction. For each slot, either shoreline or frequency matching was applied and geometrically corrected accordingly. It would be an interesting future research project to collect all shoreline matching results as GCPs and all frequency matching results as tiepoints and apply rigorous photogrammetric bundle adjustments to simultaneously model all slots. We also plan to improve the accuracy of frequency matching and eliminate outliers automatically by using a number of frequency matching and corresponding matching results.

## Figures and Tables

**Table 1:** Construction of shoreline chips.

Slot	Number of Shoreline Chips
0	5
1	6
2	41
3	52
4	58
5	27
6	62
7	50
8	31
9	30
10	46
11	18
15	25
**Total**	**451**

**Table 2:** Shoreline-matching result (before RANSAC).

Slot	Acquisition Time (UTC)															
	0		1		2		3		4		5		6		7	
	Match	Outlier	Match	Outlier	Match	Outlier	Match	Outlier	Match	Outlier	Match	Outlier	Match	Outlier	Match	Outlier
0	5	1	4	2	4	2	5	1	4	2	4	2	5	1	5	1
1	6	0	6	0	6	0	6	0	6	0	6	0	6	0	6	0
2	41	3	41	3	41	1	41	1	41	3	41	3	41	3	41	3
3	52	0	46	2	41	0	40	0	45	0	52	0	51	1	50	2
4	58	0	56	1	56	0	56	0	58	0	58	0	58	0	57	1
5	29	0	29	0	29	0	29	0	29	0	29	0	29	0	29	0
6	59	2	59	2	59	2	58	3	60	1	60	1	60	1	61	0
7	43	2	41	4	43	2	43	2	43	2	43	1	40	4	37	8
8	33	14	37	9	38	7	39	6	39	8	37	10	41	6	34	13
9	29	1	29	1	29	1	30	0	30	0	29	1	29	1	29	1
10	46	0	46	0	46	0	45	0	45	0	46	0	46	0	46	0
11	13	0	13	0	13	0	13	0	13	0	13	0	13	0	13	0
15	7	10	12	4	16	1	24	2	29	1	19	2	19	3	24	8
Total	421	33	419	28	421	16	429	15	442	17	437	20	438	20	432	37

**Table 3:** Shoreline-matching result (after RANSAC).

Slot	Acquisition Time (UTC)															
	0		1		2		3		4		5		6		7	
	Inlier	Outlier	Inlier	Outlier	Inlier	Outlier	Inlier	Outlier	Inlier	Outlier	Inlier	Outlier	Inlier	Outlier	Inlier	Outlier
0	4	0	3	0	4	0	5	0	4	0	4	0	5	0	4	0
1	6	0	6	0	6	0	6	0	6	0	6	0	6	0	6	0
2	40	0	41	0	41	0	41	0	40	0	42	0	40	0	40	0
3	47	0	43	0	37	0	37	0	42	0	47	0	47	0	45	0
4	54	0	53	0	55	0	54	0	56	0	56	0	56	0	53	0
5	28	0	29	0	28	0	28	0	29	0	28	0	28	0	28	0
6	59	0	59	0	59	0	52	0	60	0	60	0	60	0	61	0
7	41	0	41	0	42	0	41	0	40	0	40	0	39	0	39	0
8	23	0	24	0	26	0	29	0	25	0	27	0	26	0	23	0
9	29	0	29	0	29	0	30	0	30	0	29	0	29	0	29	0
10	45	0	46	0	46	0	43	0	43	0	45	0	45	0	45	0
11	13	0	13	0	13	0	13	0	13	0	13	0	13	0	13	0
15	6	0	11	0	16	0	24	0	29	0	19	0	19	0	24	0
Total	395	0	398	0	402	0	403	0	417	0	416	0	413	0	410	0

**Table 4:** RMSE of geometric correction with precision sensor modeling (in km).

Slot	Acquisition Time (UTC)								Average Error
	0	1	2	3	4	5	6	7	
0	1.8	1.9	1.4	1.0	8.4	1.4	1.7	4.1	2.7
1	1.1	1.2	1.7	1.0	1.7	1.1	1.2	0.8	1.2
2	1.2	1.1	1.0	1.2	1.2	1.0	0.8	1.1	1.1
3	1.0	0.8	1.1	0.8	0.8	0.9	1.1	1.1	0.9
4	0.9	1.0	0.9	0.9	1.1	1.1	0.9	1.0	1.0
5	1.0	0.8	0.9	0.9	0.7	0.7	0.8	0.7	0.8
6	0.6	0.6	0.6	0.7	0.6	0.8	0.7	0.7	0.7
7	0.4	0.4	0.4	0.4	0.6	0.6	0.6	0.5	0.5
8	1.1	1.1	1.0	1.1	1.5	1.0	1.3	0.8	1.1
9	1.1	0.8	1.0	1.1	0.9	0.8	0.9	0.8	0.9
10	1.0	1.1	1.0	0.9	1.0	0.9	1.0	1.0	1.0
11	1.0	1.1	0.9	1.0	1.0	0.9	1.0	1.0	1.0
15	1.0	0.8	0.9	0.8	0.9	1.0	1.0	1.0	0.9
**Average**	1.0	1.0	1.0	0.9	1.6	0.9	1.0	1.1	1.1

**Table 5:** Frequency-matching results.

Slot	Acquisition Time (UTC)															
	0		1		2		3		4		5		6		7	
	Match	Outlier	Match	Outlier	Match	Outlier	Match	Outlier	Match	Outlier	Match	Outlier	Match	Outlier	Match	Outlier
**0**	1	0	1	0	1	0	1	0	1	0	1	0	1	0	1	0
**1**	2	0	2	0	2	0	2	0	1	1	2	0	2	0	1	1
**2**	2	0	2	0	2	0	2	0	2	0	2	0	2	0	2	0
**3**	2	0	2	0	2	0	2	0	2	0	2	0	2	0	2	0
**4**	2	0	2	0	2	0	2	0	2	0	2	0	2	0	2	0
**5**	2	0	2	0	2	0	2	0	1	1	2	0	1	1	2	0
**6**	2	0	2	0	2	0	2	0	1	1	2	0	1	1	2	0
**7**	2	0	2	0	2	0	2	0	2	0	2	0	2	0	2	0
**8**	2	0	2	0	2	0	2	0	2	0	1	1	2	0	2	0
**9**	2	0	2	0	2	0	2	0	2	0	1	1	2	0	2	0
**10**	2	0	2	0	2	0	2	0	2	0	2	0	2	0	2	0
**11**	1	0	1	0	1	0	1	0	1	0	1	0	1	0	1	0
**15**	1	0	1	0	1	0	1	0	1	0	1	0	1	0	1	0
**Total**	23	0	23	0	23	0	23	0	20	3	21	2	21	2	22	1

**Table 6:** RMSE of geometric correction with frequency matching (includes outliers) (in km).

Slot	Acquisition Time (UTC)								Average Error
	0	1	2	3	4	5	6	7	
**0**	1.8	2.1	3.9	1.4	3.4	2.2	0.9	1.1	2.1
**1**	1.4	1.6	1.6	1.9	3.9	1.4	1.4	3.3	2.1
**2**	1.3	1.5	1.4	1.2	1.3	1.0	1.0	0.8	1.2
**3**	1.3	0.9	1.2	1.3	1.4	1.4	0.9	1.5	1.2
**4**	0.9	1.0	1.0	0.9	0.9	0.9	0.7	0.4	0.8
**5**	1.2	2.2	1.5	1.7	9.6	1.6	1.7	1.6	2.6
**6**	1.5	1.0	1.3	0.9	9.3	1.6	3.5	1.6	2.6
**7**	1.1	1.2	1.1	1.8	1.4	1.5	1.5	1.5	1.4
**8**	1.3	1.2	0.8	2.1	1.4	2.9	0.5	1.1	1.4
**9**	1.1	0.7	0.7	0.9	1.6	2.9	0.3	0.6	1.1
**10**	0.6	0.3	0.4	0.5	0.3	0.3	0.6	0.6	0.5
**11**	1.5	1.6	1.1	1.3	1.4	1.2	1.4	1.4	1.4
**15**	0.5	0.9	0.7	2.6	0.4	1.4	1.5	1.6	1.2
**Average**	1.2	1.2	1.3	1.4	2.8	1.5	1.2	1.3	1.5

**Table 7:** RMSE of geometric correction with frequency matching (excludes outliers) (in km).

Slot	Acquisition Time (UTC)								Average Error
	0	1	2	3	4	5	6	7	
0	1.8	2.1	3.9	1.4	3.4	2.2	0.9	1.1	2.1
1	1.4	1.6	1.6	1.9	1.5	1.4	1.4	1.4	1.5
2	1.3	1.5	1.4	1.2	1.3	1.0	1.0	0.8	1.2
3	1.3	0.9	1.2	1.3	1.4	1.4	0.9	1.5	1.2
4	0.9	1.0	1.0	0.9	0.9	0.9	0.7	0.4	0.8
5	1.2	2.2	1.5	1.7	1.1	1.6	0.9	1.6	1.5
6	1.5	1.0	1.3	0.9	0.9	1.6	0.8	1.6	1.2
7	1.1	1.2	1.1	1.8	1.4	1.5	1.5	1.5	1.4
8	1.3	1.2	0.8	2.1	1.4	1.4	0.5	1.1	1.2
9	1.1	0.7	0.7	0.9	1.6	0.5	0.3	0.6	0.8
10	0.6	0.3	0.4	0.5	0.3	0.3	0.6	0.6	0.5
11	1.5	1.6	1.1	1.3	1.4	1.2	1.4	1.4	1.4
15	0.5	0.9	0.7	2.6	0.4	1.4	1.5	1.6	1.2
**Average**	1.2	1.2	1.3	1.4	1.3	1.2	1.0	1.2	1.2
